# Mitochondrial ferritin is a functional iron-storage protein in cucumber (*Cucumis sativus*) roots

**DOI:** 10.3389/fpls.2013.00316

**Published:** 2013-08-16

**Authors:** Gianpiero Vigani, Delia Tarantino, Irene Murgia

**Affiliations:** ^1^Dipartimento di Scienze Agrarie e Ambientali – Produzione, Territorio, Agroenergia, Università degli Studi di MilanoMilano, Italy; ^2^Dipartimento di Bioscienze, Università degli Studi di MilanoMilano, Italy

**Keywords:** *Cucumis sativus*, ferritin, iron homeostasis, iron-storage protein, mitochondria, micronutrients, O_2_ consumption, roots

## Abstract

In plants, intracellular Fe trafficking must satisfy chloroplasts' and mitochondrial demands for Fe without allowing its accumulation in the organelles in dangerous redox-active forms. Protein ferritin is involved in such homeostatic control, however its functional role in mitochondria, differently from its role in chloroplasts, is still matter of debate. To test ferritin functionality as a 24-mer Fe-storage complex in mitochondria, cucumber seedlings were grown under different conditions of Fe supply (excess, control, deficiency) and mitochondria were purified from the roots. A ferritin monomer of around 25 KDa was detected by SDS-PAGE in Fe-excess root mitochondria, corresponding to the annotated Csa5M215130/XP_004163524 protein: such a monomer is barely detectable in the control mitochondria and not at all in the Fe-deficient ones. Correspondingly, the ferritin 24-mer complex is abundant in root mitochondria from Fe-excess plants and it stores Fe as Fe(III): such a complex is also detectable, though to a much smaller extent, in control mitochondria, but not in Fe-deficient ones. Cucumber ferritin Csa5M215130/XP_004163524 is therefore a functional Fe(III)-store in root mitochondria and its abundance is dependent on the Fe nutritional status of the plant.

## Introduction

The detailed understanding of molecular mechanisms regulating plant nutrient homeostasis is of the highest priority and represents one of the hundred most relevant questions facing plant research (Grierson et al., 2011). Keeping iron (Fe) homeostasis under control is particularly relevant for plants due to its essential role in many house-keeping cellular functions, but also to its toxicity as catalyst of the Fenton reaction, when in a free form (Winterbourn, 1995; Briat et al., 2010). A picture is now emerging of how the Fe deficiency responses, uptake, transport and distribution to various plant organs, together with its intracellular trafficking and storage, are finely tuned on the availability of soluble Fe in the soil by a complex net of signal transduction pathways (Vigani et al., 2009; Murgia et al., 2009; Conte and Walker, 2011; Ramirez et al., 2011; Kobayashi and Nishizawa, 2012; Vigani et al., 2013a,b; Thomine and Vert, 2013).

The intra-organellar partitioning of Fe under various nutritional conditions is intriguing: the question of whether modifications of mitochondrial and chloroplastic Fe metabolism, in response to alteration in the plant Fe status, represent a source of retrograde signals necessary to regulate the nuclear gene expression, has been posed (Vigani et al., 2013a). For that, metabolic changes in organelles occurring under various conditions of Fe supply should be documented in detail (Vigani et al., 2013a). Investigation of mitochondria from roots is particularly attractive, since the lack of photosynthetically active chloroplasts might reduce the complexity of the retrograde signals involved. Moreover, roots are directly involved in uptake of Fe from soil and their mitochondria provide chemical energy for such a process.

An extra layer of complexity involving plant Fe nutrition is given by the necessity to prevent Fe accumulation in dangerous, redox-active forms, in the organelles as well as in the cytosol. Ferritin protein, by forming a 24-mer cage-like structure able to store Fe in a safe, bioavailable form, is involved in such intracellular control of Fe trafficking and homeostasis, in both plant and animal cells (Arosio et al., 2009; Briat et al., 2009). In plants, ferritin is targeted to chloroplasts (Briat et al., 2009) but its localization to mitochondria has been also documented in Arabidopsis and in pea (Zancani et al., 2004; Tarantino et al., 2010a,b).

The characterization of Arabidopsis *atfer4* mutants knock-out for the ferritin isoform, targeted also to mitochondria (Tarantino et al., 2010a,b), posed the question of whether the role of the mitochondrial-targeted AtFER4 is a sort of ancestral relict, replaced by other still unknown regulatory mechanisms of Fe homeostasis, during the evolution to green plants. On the other side, a relevant role of human mitochondrial ferritin (Levi et al., 2001) as protectant against oxidative stress in various cell types (Campanella et al., 2009; Wang et al., 2011) but also in improving respiratory function in yeast mutants deficient in [Fe-S] cluster biogenesis (Sutak et al., 2012), is emerging.

Taken together, this evidence prompted us to investigate whether ferritin is functional in the mitochondria of cucumber (*Cucumis sativus*) roots, that is, if it can truly store Fe(III), as a 24-mer complex.

## Materials and methods

### Plant growth conditions and purification of root mitochondria

Seeds of cucumber (*Cucumi sativus* L. cv. Marketmore) were sown in Agriperlite, watered with 0.1 mM CaSO_4_, allowed to germinate in the dark at 26°C for 3 d, and then transferred to a nutrient solution with the following composition: 2 mM Ca(NO)_3_, 0.75 mM K_2_SO_4_, 0.65 mM MgSO_4_, 0.5 mM KH_2_PO_4_, 10 μM H_3_BO_3_, 1 μM MnSO_4_, 0.5 μM CuSO_4_, 0.5 μM ZnSO_4_, 0.05 μM (NH_4_)Mo_7_O_24_, and Fe(III)-EDTA at the following concentrations: 0 mM (Fe deficiency), 0.05 mM (Control), 0.5 mM (Fe excess). The pH was adjusted to 6.0–6.2 with NaOH. Aerated hydroponic cultures were maintained in a growth chamber with a day: night regime of 16:8 h and 200 μE m^−2^s^−1^ photosynthetically active radiation (PAR) at the plant level. The temperature ranged from 18°C (in the dark) to 24°C (in the light).

Mitochondria were purified from cucumber roots according to Balk et al. (1999) and Vigani et al. (2009), with few modifications. 10 days old roots were homogenized with a mortar and pestle in 0.4 M mannitol, 25 mM MOPS pH 7.8, 1 mM EGTA, 8 mM cysteine, and 0.1% (w/v) bovine serum albumin (BSA). The filtered homogenized plant material (total extract, TE) was centrifuged 5 min at 4000 g and the pellet was used as an enriched plastids fraction (P). The supernatant was re-centrifuged 15 min at 12,000 g to pellet mitochondria whereas the supernatant fraction constitutes the so-called PMS (post-mitochondria supernatant fraction). The crude mitochondrial pellet was resuspended in RB buffer (0.4 M mannitol, 10 mM Tricine pH 7.2, 1 mM EGTA) and lightly homogenized with a potter; mitochondria were further purified on a 40, 28, and 13.5% (v/v) Percoll (Pharmacia) step gradient in RB buffer. The fraction at the 28/40% interface (purified mitochondria) was collected and washed by differential centrifugation in RB buffer.

### Native gel electrophoresis and western blot analysis

Purified mitochondrial proteins were loaded on a non-denaturing polyacrylamide gel (3% [w/v] stacking, 5.5% [w/v] separating) after heating at 65°C for 7 min. The gel was run for 5 h at 25 mA (on ice); then the gel was rinsed in water and incubated in potassium ferrous cyanide solution [2% KFe(II)CN, 2% HCl], 1 h in the dark. The gel was washed four times in H_2_O, 15 min each, with gentle shaking and incubated overnight in a diaminobenzidine (DAB) solution (0.05% DAB, 18 mM H_2_O_2_ in PBS at pH 7.4) without shaking.

SDS-PAGE was performed according to Vigani et al. (2009) with the following antibodies: spinach anti-Toc33 (Rödiger et al., 2010) at 1:1000 dilution, maize anti-porin (Balk and Leaver, 2001) at 1:2000 dilution, Arabidopsis anti-ATFER1 (Murgia et al., 2007) at 1:2000 and an anti-rabbit conjugated with alkaline phosphatase as secondary antibody. Protein quantification was determined according to Vigani et al. (2009).

### RT-PCR

Roots apices and true second leaves from 10 days old plants grown under Fe-excess were sampled and RNA extracted with Trizol reagent (Gibco). RT-PCR reactions were performed by using Access RT-PCR kit (Promega), 60 ng total RNA/reaction.

Cucsafor1:5′-CCACCACACACACACACGC-3′
Cucsarev1:5′-ATTGTCTCTGTCAAAGTAGGC-3′
Cucsarev2: 5′-TTGGCCAAACCCTTGAGTGC-3′
Cucsarev3: 5′-CCATTGCAAAAAAAGCATCTCC-3′
Cucsarev4: 5′-GAGCTCCATTGCATATAAGGC-3′

For Cucsafor1-Cucsarev1: 1 mM MgSO_4_ final conc., annealing at 61°C; for Cucsafor1-Cucsarev2: 1.5 mM MgSO_4_ final conc., annealing at 61°C; for Cucsafor1-Cucsarev3: 1.5 mM MgSO_4_ final conc., annealing at 57–67°C; for Cucsafor1-Cucsarev4: 1 mM MgSO_4_ final conc., annealing at 61–67°C.

### Miscellanous

Total Chl, Chla and Chlb content was determined according to Lichtenthaler (1987). Fe content in purified mitochondria was determined by ICP-MS spectroscopy (Variant).

F_0_F_1_ATP synthase and G6PDH activities were performed according to Camacho-Pereira et al. (2009).

O_2_ consumption and use of KCN and SHAM (salicylhydroxamic acid) was measured on root apices from 10 days-old plants according to Vigani et al. (2009).

Protein sequence alignment was performed with Multalin version 5.4.1 (Corpet, 1988) at http://multalin.toulouse.inra.fr/multalin/.

## Results

### Fe-deficient and fe-excess root tips show an increase in the O_2_ consumption rates

**Figure 1:**
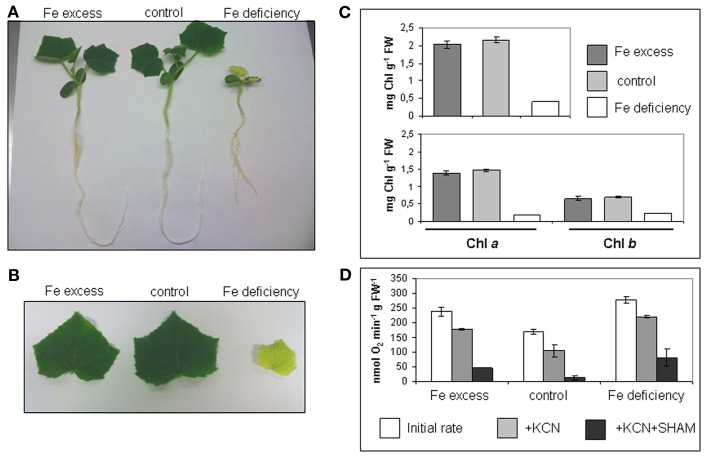
**Cucumber seedlings under different conditions of Fe supply. (A)** 10 days-old cucumber seedlings grown in hydroponic medium containing either 500 μM Fe(III)-EDTA (Fe excess), 50 μM Fe(III)-EDTA (control), or with no added Fe in the medium (Fe deficiency). **(B)** Leaves of cucumber seedlings grown as in **(A)**. **(C)** Chl content (mg g^−1^ FW) of seedlings leaves, grown as in **(A)**; upper panel: total Chl content; lower panel: Chl*a* and Chl*b* content. Bars represent mean values ± SE from 4 independent samples of 3–4 leaves each **(D)** O_2_ consumption in seedlings roots, grown as in **(A)**; for each condition (Fe excess, control, Fe deficiency), the initial rate and the rates after addition of KCN or KCN + SHAM are provided. Bars represent mean values ± SE from 6 independent samples of 10 root tips each.

Cucumber seedlings were grown in hydroponic medium for 10 days in a complete nutrient solution containing 0, 50, or 500 μM Fe(III)-EDTA (Fe deficiency, control, excess) (Figure 1A). Fe deficient plants showed the typical symptoms of chlorosis in leaves, while leaves from both control or Fe excess plants were green; length of root apparatus was reduced in Fe-deficient plants (Figures 1A,B). Accordingly, total Chl content was very low in the Fe-deficient leaves (Figure 1C), while no significant differences in total Chl nor in Chla/Chlb content, could be detected between Fe-excess and control leaves (Figure 1C).

Root tips are the most metabolically active parts of the roots and indeed they are the main site displaying Fe uptake in which Strategy I activities are strongly induced (Landsberg, 1986; Vigani et al., 2012) Both Fe-deficient and Fe-excess root tips show higher O_2_ consumption rates than control root tips (Figure 1D). Moreover, the addition of KCN and SHAM (inhibitors of the respiratory O_2_ consumption) did not completely block the O_2_ consumption, differently from what was observed in control root tips where such inhibitors almost completely abolished it (Figure 1D). Such residual O_2_ consumption in root tissues of Fe deficient as well as of Fe-excess plants, not attributable to the mitochondrial respiratory chain activity, has been already described in cucumber (Vigani et al., 2009).

### Purity of mitochondria isolated from cucumber roots

**Figure 2:**
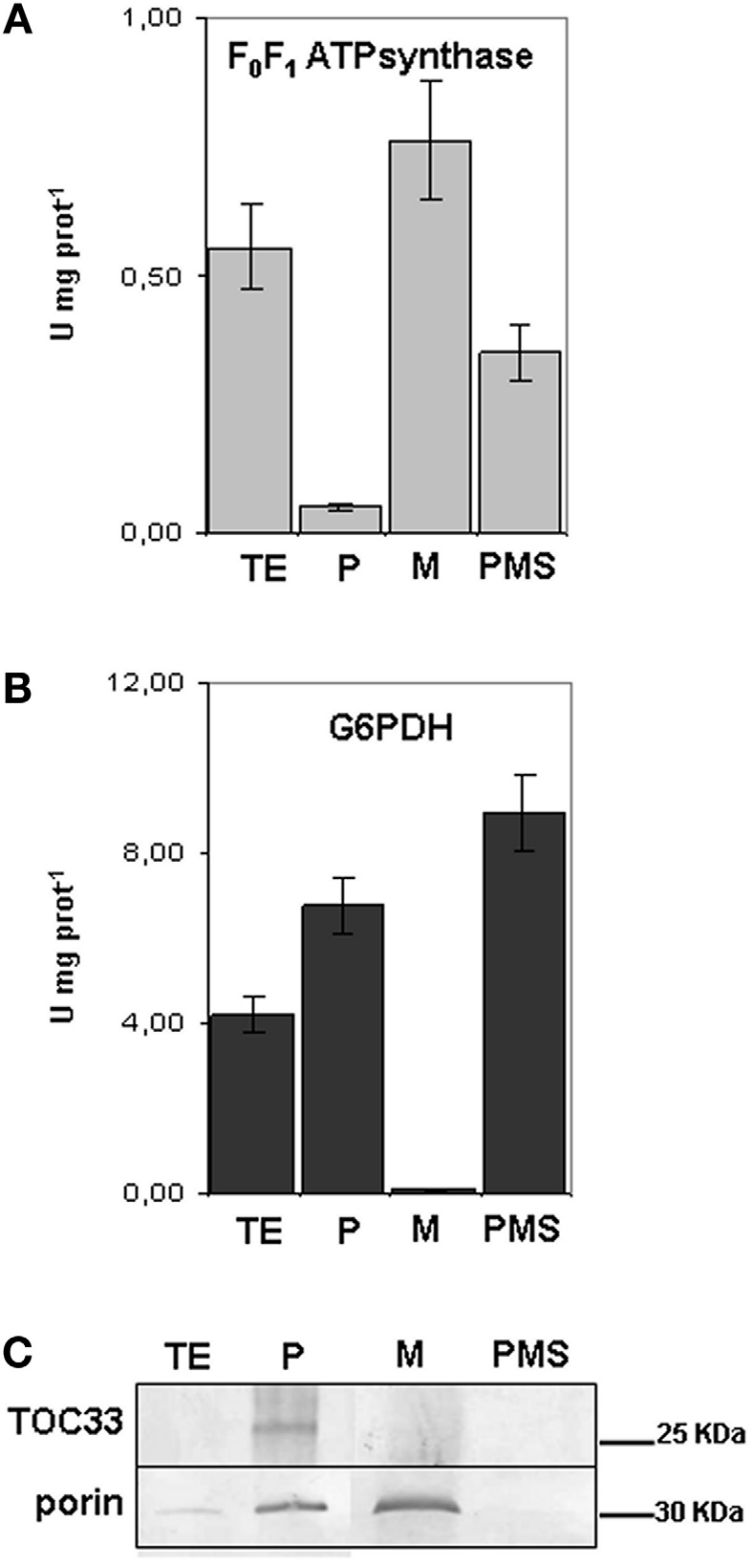
**Purity of mitochondria isolated from cucumber roots. (A)** F_0_F_1_ATPsynthase activity, expressed as Units mg^−1^ protein in the different fractions obtained during purification of mitochondria: total extract (TE), enriched plastids (P), mitochondria (M) and the post- mitochondria supernatant fraction (PMS). Bars represent the mean values ± SE from 4 independent samples of 30–50 μg protein each. **(B)** Glucose 6-phosphate dehydrogenase activity (G6PDH) in TE, P, M and PMS fraction. Bars represent the mean values ± SE from 4 independent samples of 30–50 μg protein each. **(C)** SDS-PAGE, followed by western blot analysis, of TE, P, M and PMS fractions; equal protein quantity (12 μg) was loaded in each lane.

A one step-gradient protocol was applied for purifying mitochondria from seedling roots grown in the different conditions of Fe supply. Since it is well established that ferritin accumulates in plastids of plants grown under Fe-excess, all the different fractions collected during isolation of mitochondria from Fe-excess roots, i.e., the TE, the plastid enriched fraction (P), the purified mitochondria (M) and the post-mitochondrial supernatant (PMS) (Balk et al., 2004) were tested with two different enzymatic assays as well as with different antibodies (in SDS-PAGE experiments), in order to exclude any contamination of the purified mitochondrial fractions with intact plastids. Activity of F_0_F_1_ATP synthase enzyme, a marker used for mitochondria (Camacho-Pereira et al., 2009) was measured in the four isolated fractions TE, P, M, and PMS (Figure 2A). F_0_F_1_ATP synthase activity is highest in the M fraction (0.79 U mg^−1^ prot), and, as expected, also in the TE fraction (0.56 U mg^−1^ prot) (Figure 2A). A 40% reduction of enzyme activity is observed in PMS fraction containing the cytosol (0.35 U mg^−1^prot) whereas it is almost undetectable in the P fraction (0.05 U mg^−1^ prot) (Figure 2A). Glucose 6 phosphate dehydrogenase (G6PDH) is localized both in cytosol and in the plastids' stroma (Camacho-Pereira et al., 2009) therefore it can be used as a cytosolic and plastidial enzyme marker; its activity is undetectable in M fraction (Figure 2B), whereas it is highest in the PMS (8.95 U mg^−1^prot), followed by P (6.70 U mg^−1^prot), and TE (4.20 U mg^−1^ prot) (Figure 2B). The two enzyme assays confirmed that the purified mitochondria are not contaminated by the cytosolic fraction nor from integral, undisrupted plastids, since in that case the G6PDH enzymatic activity should have been detected also in the mitochondrial fraction. Furthermore, the plastid marker Toc33 protein (Rödiger et al., 2010) accumulated only in the P fraction (Figure 2C), whereas porin (Balk and Leaver, 2001) accumulated, as expected, in the M fraction and, to lesser extent, in the P fraction (Figure 2C).

### A ferritin 24-mer complex accumulates in the root mitochondria from fe-excess plants and it stores fe(III)

**Figure 3:**
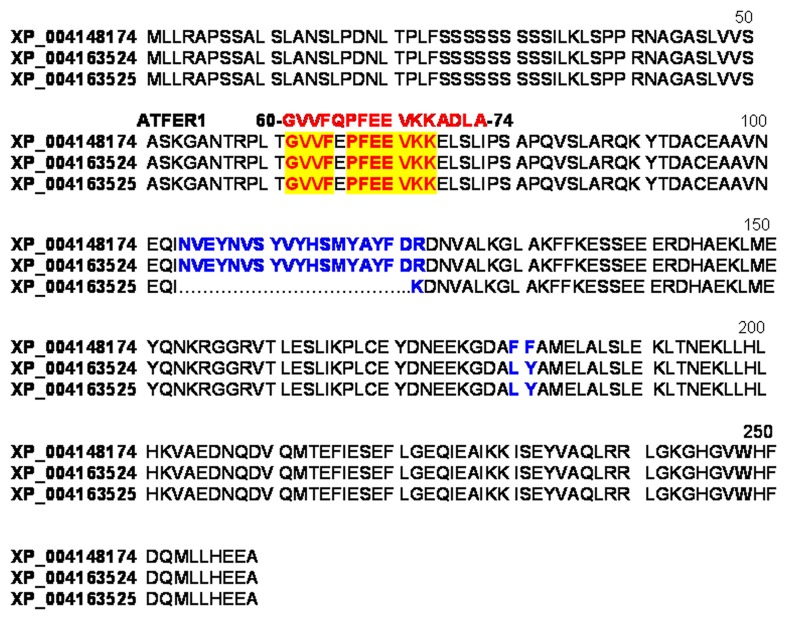
**Sequence alignment of the three predicted cucumber ferritin protein variants of the unique ferritin gene: XP_004148174, XP_004163524 identical to Csa5M215130 and XP_004163525**. In blue are the aa residues differing among the three sequences; highlighted in red are the aa identical to those in the 16-aa peptide GVVFQPFEEVKKADL used as antigen for raising the anti-ATFER1 antibody.

The genome of the Chinese Long cultivar of cucumber (line 9930) was first annotated in 2009 and then reassembled and reannotated (Huang et al., 2009; Li et al., 2011) (www.icugi.org/cgi-bin/ICuGI/index.cgi). According to that annotation, a unique ferritin protein sequence, named Csa5M215130 (259 aa), is encoded by the cucumber genome (Figure 3). The genome of a North-European cultivar (B10) has been also sequenced (Wóycicki et al., 2011) and again, a unique ferritin gene has been identified, LOC101221012 with three different transcript variants, the protein sequences of which are deposited in the NCBI database: XP_004148174 (259 aa), XP_004163524 identical to Csa5M215130 (259 aa) and XP_004163525 (241 aa) (Figure 3). XP_004148174 and XP_004163524 differ by two aa at position 180–181 (Figure 3), whereas XP_004163525 lacks a 18 aa stretch at position 104 (Figure 3). A further sequencing of the cucumber genome has been made available at Phytozome (http://www.phytozome.net), a comparative hub for plant genome analysis (Goodstein et al., 2012). According to this, the cucumber genome possesses a single ferritin gene Cucsa144440 with its primary transcript coding for a protein identical to Csa5M215130/XP_004163524.

To investigate ferritin localization and its physiological role in root mitochondria, quantification of Fe content, by ICP-MS analysis, in mitochondria purified from roots of plants grown under different conditions of Fe supply, was first performed.

**Figure 4:**
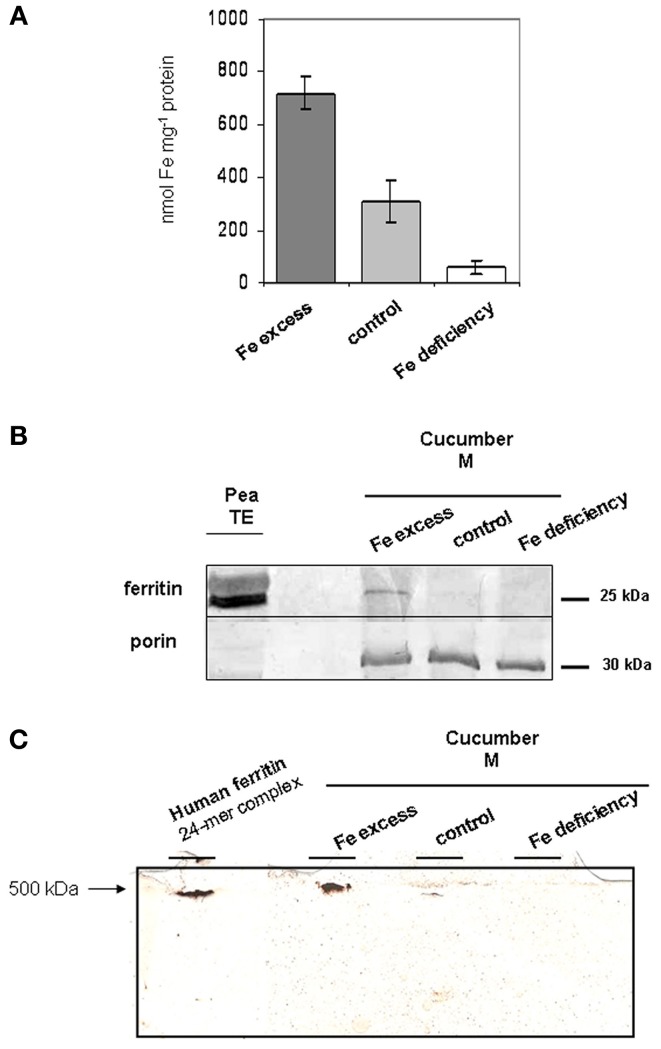
**Fe and ferritin content in mitochondria from cucumber roots grown under different conditions of Fe supply. (A)** Total Fe content in mitochondria purified from Fe-excess, control, or Fe-deficiency roots, quantified by ICP-MS. Each bar represents the mean value ± SE from 4 independent samples consisting of 60–200 μg mitochondria each. **(B)** SDS-PAGE and western blot analysis of mitochondria purified from Fe-excess, control, or Fe-deficiency root. 15 μg proteins were loaded in each lane. As positive control, 34 μg total extract (TE) from pea seeds, rich in ferritin, has been loaded in leftmost lane. **(C)** native PAGE analysis, followed by Prussian blue/DAB stain of mitochondria purified from Fe-excess, control or Fe-deficiency root; 40 μg proteins were loaded in each lane. As positive control, 48 μg proteins recombinant human ferritin consisting of a 24-mer of around 500 KDa, was also loaded in the far left lane.

Total Fe in mitochondria from Fe-excess roots is 718.92 nmol mg^−1^ prot, more than two-fold higher than in control ones (309.77 nmol mg^−1^ prot) (Figure 4A); this result confirms that growth of cucumber plants under Fe-excess treatment is effective in perturbing the Fe content of their mitochondria, beside their function, such as the described increase in O_2_ consumption (Figure 1D). Total Fe content is, instead, dramatically reduced in mitochondria purified from Fe-deficient plants; nevertheless, such Fe-deficient mitochondria are still able to perform their respiratory function (Figure 1D) thus suggesting that mitochondria are still functional in such stress conditions.

The mitochondria purified from control, Fe-excess or Fe-deficient roots were then analyzed by western blot with the Arabidopsis anti-ATFER1 ferritin antibody (Murgia et al., 2007), raised against the 16-aa antigen peptide GVVFQPFEEVKKADL corresponding to aa 60–74 in the Arabidopsis ATFER1 sequence; such an antibody is appropriate for the detection of cucumber ferritin since both Csa5M215130/XP_004163524, as well as XP_004148174 and XP_004163525, would show an 11/16 aa match with such a peptide (Figure 3).

As a positive control, crude protein TE from pea seeds (rich in ferritin) was used (Figure 4B); pea ferritin is indeed detected by the anti-ATFER1 antibody (Murgia et al., 2007).

A single band of around 25 KDa could be detected, the accumulation of which is dependent on Fe-content of the mitochondria, being strong in Fe-excess mitochondria, still detectable in control mitochondria and undetectable in Fe-deficient ones (Figure 4B). Equal loading of protein content for each sample was confirmed through hybridization with anti-porin antibody (Figure 4B) (Vigani and Zocchi, 2010).

The Fe-excess, control and Fe-deficient mitochondria, tested above, were also analyzed by native gel electrophoresis followed by Prussian blue staining, which stains Fe in the oxidized form Fe(III), and DAB/H_2_O_2_ enhancement (Luscieti et al., 2010). Recombinant human ferritin 24-mer complex (around 500 kDa) served as a positive control. A band of higher molecular weight than the recombinant human ferritin complex is clearly detected in Fe-excess mitochondria; such band is fairly weak in control mitochondria and undetectable in Fe-deficient ones (Figure 4C).

**Figure 5:**
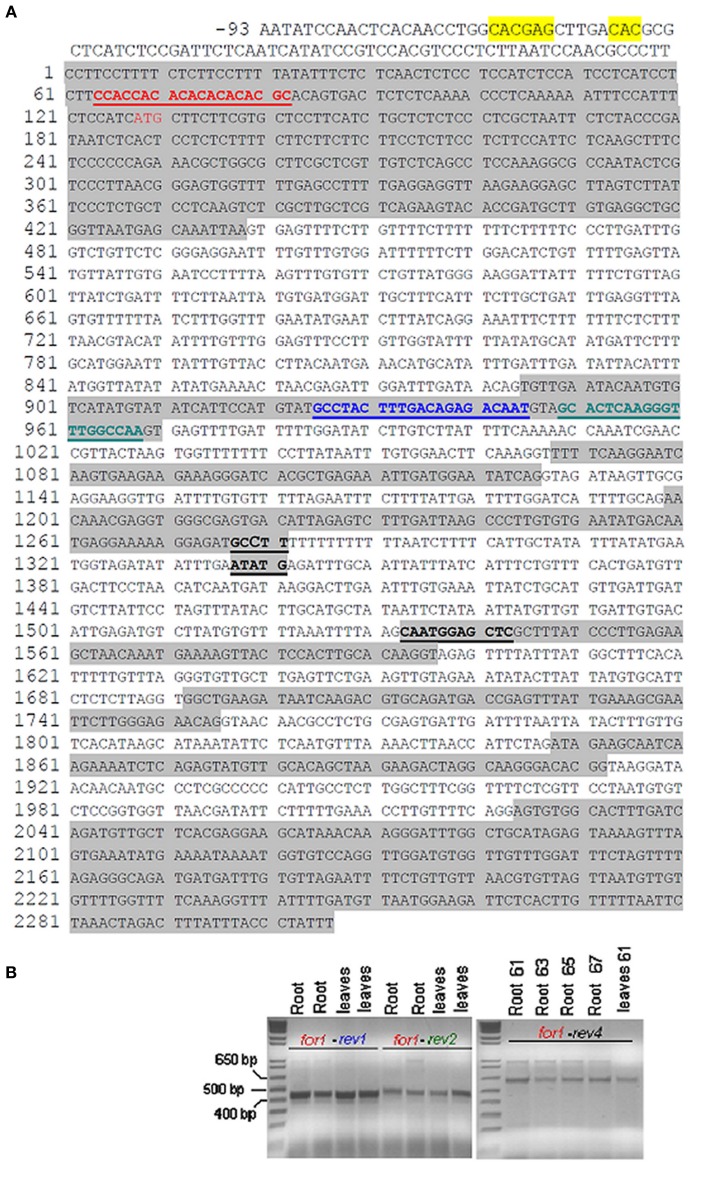
**Gene and predicted coding sequence of cucumber ferritin protein XP004163524 (reported at NCBI as ferritin-3, chloroplastic-like, transcript variant 1, LOC101221012). (A)** IDRS-like sequence in gene promoter is highlighted in yellow; ATG start is in red color; exons are highlighted in gray. Positions of primers Cucsafor1 (in red), Cucsarev1 (in blue), Cucsarev2 (in green), Cucsarev4 (in black) are underlined; **(B)** left panel: RT-PCR reactions with primer pairs Cucsafor1-Cucsa-rev1 and Cucsafor1-Cucsa-rev2, with either two independent RNA extracts from Fe-excess cucumber roots or two independent RNA extracts from Fe-excess leaves. The Kb ladder Plus (Invitrogen) has been loaded in the far left lane. Right panel: RT-PCR reactions with primer pair Cucsafor1-Cucsa-rev4, with RNA purified from Fe-excess roots at 61, 63, 65, 67 anneal. temp., or with RNA purified from Fe-excess leaves at 61°C anneal. temp.

Identity of the ferritin detected in Fe-excess mitochondria (Figures 4B,C) could be unambiguously attributed to Csa5M215130/XP_004163524. Indeed, based on gene and predicted coding sequences of XP004163524 (annotated at NCBI as ferritin-3, chloroplast-like, transcript variant 1 (LOC101221012) (Figure 5), of XP004163525 (annotated at NCBI as ferritin-3, chloroplast-like, transcript variant 2) (Figure S1) and of XP_004148174 (annotated at NCBI as ferritin-3, chloroplast-like) (Figure S2) five different primers Cucsafor1, Cucsarev1-rev4 were designed for RT-PCR experiments with RNA purified from either roots or leaves from Fe-excess cucumber plants (thus accumulating ferritin). Results show that RT-PCR with the following pairs: Cucsafor1-Cucsarev1 and Cucsafor1-Cucsarev2, amplified, respectively a fragment of 436 and 470 bp (Figure 5B, left panel), as expected from an mRNA coding for XP004163524 (Figure 5A) but not from an mRNA coding for XP004163525 (Figure S1). Moreover, RT-PCR with the Cucsafor1-Cucsarev4 primer pair amplified a fragment of 612 bp (Figure 5B, right panel), expected from an mRNA coding for XP004163524 (Figure 5A) but not from an mRNA coding for XP_004148174 (Figure S2); amplification of such fragment, in roots and in leaves, has been obtained not only at the optimal annealing temperature for that primer pair (61°C), but also at more stringent conditions, i.e., with annealing temperatures up to 67°C (Figure 5B, right panel). Viceversa, RT-PCR with Cucsafor1-Cucsarev3 primer pair (Figure S2) performed with RNA extractions from different roots and leaf samples, could not amplify any fragment expected from the mRNA coding for XP_004148174 (data not shown).

The transcription of a single mRNA coding for cucumber ferritin in roots as well as in green leaves with consequent translation of a unique ferritin protein, strongly suggests that cucumber ferritin is “dual targeted” in both mitochondria and chloroplasts (Carrie and Small, 2013). PSort (http://psort.hgc.jp/form2.html), MitoProtII (http://ihg.gsf.de/ihg/mitoprot.html) and TargetP (http://www.cbs.dtu.dk/services/TargetP/) programs are three widely used bioinformatic tools predicting subcellular localization of a given protein sequence and cleavage site of the corresponding transit peptide (Nakai and Kanehisa, 1991; Claros and Vincens, 1996; Emanuelsson et al., 2007). Such programs, when applied to the three putative protein variants Csa5M215130/XP_004163524, XP_004148174 and XP_004163525, for prediciting the mitochondrial localization, give different results: MitoProtII scores are indeed, respectively: 0.82, 0.79, 0.80; PSort scores are 0.47 for all the three sequences whereas TargetP scores are below 0.1 for all the three sequences. The predictions of the cleavage site for the transit peptide are also quite different among the three programs, being of 68 aa with MitoProtII, 32 aa with PSort and 50 aa with TargetP. Variability of such results is not surprising, since it is widely known that such bioinformatic predictors proved extremely problematic for dual targeted proteins (Pujol et al., 2007; Berglund et al., 2009; Carrie and Small, 2013).

A 14 bp Iron Dependent Regulatory Sequence (IDRS) is present in the promoter region of the Arabidopsis AtFer1 gene and is responsible for the AtFer1 transcriptional repression under low Fe supply. On the contrary, Fe treatment releases the IDRS-mediated transcriptional repression of the AtFer1 gene via a NO-dependent mechanism, and AtFer1 ferritin transcript can therefore accumulate (Petit et al., 2001; Murgia et al., 2002; Arnaud et al., 2006).

A 14-bp sequence, 64% identical to the AtFer1 IDRS, is present in the promoter region of the cucumber ferritin gene LOC101221012/Csa5M215130/Cucsa144440, at position -199 from ATG start (Figure 5A), similarly to what is observed for the AtFer1 IDRS sequence (Petit et al., 2001).

## Discussion

The scientific relevance in focusing on plant nutrients and their homeostasis is not confined to the potential long-term benefits in agriculture: indeed, a better understanding of Fe homeostasis can have positive impacts and can offer new solutions for combatting malnutrition (Grierson et al., 2011). Such knowledge can assist the exploitation of new strategies for the production of plants biofortified for Fe and for reducing Fe deficiency anaemia, a severe burden for populations in developing countries mostly affecting children and women (Murgia et al., 2012).

Elucidation of the role of mitochondria in plant Fe homeostasis is a challenging issue (Vigani et al., 2013a). Despite the fact that mitochondria are strongly affected by Fe deficiency, they in fact display a high level of functional flexibility, which allows them to guarantee cell viability under Fe shortage (Vigani, 2012).

The present work confirms the localization of the ferritin in mitochondria of cucumber roots, as already observed in Arabidopsis and pea (Zancani et al., 2004; Tarantino et al., 2010a,b); its abundance is strictly dependent on the total Fe content in mitochondria which, in turn, is dependent on the availability of Fe in the growth medium.

A unique mRNA transcribed from LOC101221012 cucumber gene, coding for the ferritin protein named alternatively Csa5M215130 or XP_004163524 by two different sequencing consortia (Li et al., 2011; Wóycicki et al., 2011) has been identified in Fe-excess cucumber roots and leaves; moreover, such mRNA is 100% identical to the primary transcript of the ferritin gene named Cucsa144440 in the Phytozome hub (Goodstein et al., 2012). Results in the present work show that a single Fe-dependent ferritin isoform, named Csa5M215130/XP_004163524 accumulates in cucumber roots. Since no other transcripts are detected in green leaves of Fe-excess cucumber plants, such ferritin protein is most probably dual- targeted to both chloroplasts and mitochondria and represents an example of the so-called “ambiguous targeting” (Peeters and Small, 2001; Carrie and Small, 2013).

The presence, in LOC101221012/Cucsa144440 gene promoter ferritin, of an IDRS-like stretch, is suggestive of a common regulatory mechanism for the Fe-dependent expression of the two ferritin genes in Arabidopsis and in cucumber.

The results shown in the present work strongly suggest that the detected multimer complex is truly the 24-mer ferritin complex Csa5M215130/XP_004163524; indeed, as observed for the monomer, the abundance of the multimer complex is also dependent on the mitochondrial Fe content and results obtained in native gel are consistent with its expected weight (around 600 KDa).

In conclusion, for the first time proof that ferritin is a functional Fe-storage protein in cucumber mitochondria is provided: the 24-mer ferritin complex indeed truly binds Fe(III).

Such results open the way to further investigations about the possible physiological relevance of ferritin as a root protectant against various oxidative stresses which may arise during adverse field conditions.

### Conflict of interest statement

The authors declare that the research was conducted in the absence of any commercial or financial relationships that could be construed as a potential conflict of interest.

## Supplementary material

Figure S1 **Predicted coding sequence for XP004163525 protein**.

Figure S2 **Predicted coding sequence for XP_004148174 protein**.
