# Supplementary material for: Mitochondrial ferritin is a functional iron-storage protein in cucumber (Cucumis sativus) roots
Source: Front Plant Sci. 2013 Aug 16;4:316. doi: 10.3389/fpls.2013.00316 (PMC3744851; doi:10.3389/fpls.2013.00316)
Supplement: Figure S1 — Predicted coding sequence for XP004163525 protein. [file DataSheet1.PDF]

1 CCTTCCTTTT CTCTTCCTTT TATATTTCTC TCAACTCTCC TCCATCTCCA TCCTCATCCT

61 CTTCCACCAC ACACACACAC GCACAGTGAC TCTCTCAAAA CCCTCAAAAA ATTTCCATTT

121 CTCCATCATG CTTCTTCGTG CTCCTTCATC TGCTCTCTCC CTCGCTAATT CTCTACCCGA

181 TAATCTCACT CCTCTCTTTT CTTCTTCTTC TTCCTCTTCC TCTTCCATTC TCAAGCTTTC

241 TCCCCCCAGA AACGCTGGCG CTTCGCTCGT TGTCTCAGCC TCCAAAGGCG CCAATACTCG

301 TCCCTTAACG GGAGTGGTTT TTGAGCCTTT TGAGGAGGTT AAGAAGGAGC TTAGTCTTAT

361 TCCCTCTGCT CCTCAAGTCT CGCTTGCTCG TCAGAAGTAC ACCGATGCTT GTGAGGCTGC

421 GGTTAATGAG CAAATTAAGT GAGTTTTCTT GTTTTCTTTT TTTCTTTTTC CCTTGATTTG

481 GTCTGTTCTC GGGAGGAATT TTGTTTGTGG ATTTTTTTCTT GGACATCTGT TTTTGAGTTA

541 TGTTATTGTG AATCCTTTTA AGTTTGTGTT CTGTTATGGG AAGGATTATT TTTCTGTTAG

601 TTATCTGATT TTCTTAATTA TGTGATGGAT TGCTTTCATT TCTTGCTGAT TTGAGGTTTA

661 GTGTTTTTTA TCTTTGGTTT GAATATGAAT CTTTATCAGG AAATTTCTTT TTTTCTCTTT

721 TAACGTACAT ATTTTGTTTG GAGTTTCCTT GTTGGTATTT TTATATGCAT ATGATTCTTT

781 GCATGGAATT TATTTGTTAC CTTACAATGA AACATGCATA TTTGATTTGA TATTACATTT

841 ATGGTTATAT ATATGAAAAC TAACGAGATT GGATTTGATA ACAGTGTTGA ATACAATGTG

901 TCATATGTAT ATCATTCCAT GTATGCCTAC TTTGACAGAG ACAATGTAGC ACTCAAGGGT

961 TTGGCCAAGT GAGTTTTGAT TTTTGGATAT CTTGTCTTAT TTTCAAAAAC CAAATCGAAC

1021 CGTTACTAAG TGGTTTTTTT CCTTATAATT TGTGGAACTT CAAAGGTTTT TCAAGGAATC

1081 AAGTGAAGAA GAAAGGGATC ACGCTGAGAA ATTGATGGAA TATCAGGTAG ATAAGTTGCG

1141 AGGAAGGTTG ATTTTGTGTT TTTAGAATTT CTTTTATTGA TTTTGGATCA TTTTGCAGAA

1201 CAAACGAGGT GGGCGAGTGA CATTAGAGTC TTTGATTAAG CCCTTGTGTG AATATGACAA

1261 TGAGGAAAAA GGAGAT**GCCT T**TTTTTTTTT TTAATCTTTT CATTGCTATA TTTATATGAA

1321 TGGTAGATAT ATTTGA**ATAT G**AGATTTGCA ATTATTTATC ATTTCTGTTT CACTGATGTT

1381 GACTTCCTAA CATCAATGAT AAGGACTTGA ATTTGTGAAA TTATCTGCAT GTTGATTGAT

1441 GTCTTATTCC TAGTTTATAC TTGCATGCTA TAATTCTATA ATTATGTTGT TGATTGTGAC

1501 ATTGAGATGT CTTATGTGTT TTAAATTTTA AG**CAATGGAG CTC**GCTTTAT CCCTTGAGAA

1561 GCTAACAAAT GAAAAGTTAC TCCACTTGCA CAAGGTAGAG TTTTATTTAT GGCTTTCACA

1621 TTTTTGTTTA GGGTGTTGCT TGAGTTCTGA AGTTGTAGAA ATATACTTAT TATGTGCATT

1681 CTCTCTTAGG TGGCTGAAGA TAATCAAGAC GTGCAGATGA CCGAGTTTAT TGAAAGCGAA

1741 TTCTTGGGAG AACAGGTAAC AACGCCTCTG CGAGTGATTG ATTTTAATTA TACTTTGTTG

1801 TCACATAAGC ATAAATATTC TCAATGTTTA AAACTTAACC ATTCTAGATA GAAGCAATCA

1861 AGAAAATCTC AGAGTATGTT GCACAGCTAA GAAGACTAGG CAAGGGACAC GGTAAGGATA

1921 ACAACAATGC CCTCGCCCCC CATTGCCTCT TGGCTTTCGG TTTTCTCGTT CCTAATGTGT

1981 CTCCGGTGGT TAACGATATT CTTTTTGAAA CCTTGTTTTC AGGAGTGTGG CACTTTGATC

2041 AGATGTTGCT TCACGAGGAA GCATAAACAA AGGGATTTGG CTGCATAGAG TAAAAGTTTA

2101 GTGAAATATG AAAATAAAAT GGTGTCCAGG TTGGATGTGG TTGTTTGGAT TTCTAGTTTT

2161 AGAGGGCAGA TGATGATTTG TGTTAGAATT TTCTGTTGTT AACGTGTTAG TTAATGTTGT

2221 GTTTTGGTTT TCAAAGGTTT ATTTTGATGT TAATGGAAGA TTCTCACTTG TTTTTAATTC

2281 TAAACTAGAC TTTATTTACC CTATTT

Figure S1

Predicted coding sequence of *Cucumis sativus* ferritin protein XP004163525 (reported at NCBI as ferritin-3, chloroplastic-like, transcript variant 2 (LOC101221012). Exons are highlighted in yellow; position of primers Cucsafor1, Cucsarev1 and Cucsarev2 is underlined; position of primer Cucsarev4 is underlined in bold.

1 CCTTCCTTTT CTCTTCCTTT TATATTTCTC TCAACTCTCC TCCATCTCCA TCCTCATCCT
61 CTTCCACCAC ACACACACAC GCACAGTGAC TCTCTCAAAA CCCTCAAAAA ATTTCCATTT
121 CTCCATCATG CTTCTTCGTG CTCCTTCATC TGCTCTCTCC CTCGCTAATT CTCTACCCGA
181 TAATCTCACT CCTCTCTTTT CTTCTTCTTC TTCCTCTTCC TCTTCCATTC TCAAGCTTTC
241 TCCCCCCAGA AACGCTGGCG CTTCGCTCGT TGTCTCAGCC TCCAAAGGCG CCAATACTCG
301 TCCCTTAACG GGAGTGGTTT TTGAGCCTTT TGAGGAGGTT AAGAAGGAGC TTAGTCTTAT
361 TCCCTCTGCT CCTCAAGTCT CGCTTGCTCG TCAGAAGTAC ACCGATGCTT GTGAGGCTGC
421 GGTTAATGAG CAAATTAAGT GAGTTTTCTT GTTTTCTTTT TTTCTTTTTC CCTTGATTTG
481 GTCTGTTCTC GGGAGGAATT TTGTTTGTGG ATTTTTTCTT GGACATCTGT TTTTGAGTTA
541 TGTTATTGTG AATCCTTTTA AGTTTGTGTT CTGTTATGGG AAGGATTATT TTTCTGTTAG
601 TTATCTGATT TTCTTAATTA TGTGATGGAT TGCTTTCATT TCTTGCTGAT TTGAGGTTTA
661 GTGTTTTTTA TCTTTGGTTT GAATATGAAT CTTTATCAGG AAATTTCTTT TTTTCTCTTT
721 TAACGTACAT ATTTTGTTTG GAGTTTCCTT GTTGGTATTT TTATATGCAT ATGATTCTTT
781 GCATGGAATT TATTTGTTAC CTTACAATGA AACATGCATA TTTGATTTGA TATTACATTT
841 ATGGTTATAT ATATGAAAAC TAACGAGATT GGATTTGATA ACAGTGTTGA ATACAATGTG
901 TCATATGTAT ATCATTCCAT GTATGCCTAC TTTGACAGAG ACAATGTAGC ACTCAAGGGT
961 TTGGCCAAGT GAGTTTTGAT TTTTGGATAT CTTGTCTTAT TTTCAAAAAC CAAATCGAAC
1021 CGTTACTAAG TGGTTTTTTT CCTTATAATT TGTGGAACTT CAAAGGTTTT TCAAGGAATC
1081 AAGTGAAGAA GAAAGGGATC ACGCTGAGAA ATTGATGGAA TATCAGGTAG ATAAGTTGCG
1141 AGGAAGGTTG ATTTTGTGTT TTTAGAATTT CTTTTATTGA TTTTGGATCA TTTTGCAGAA
1201 CAAACGAGGT GGGCGAGTGA CATTAGAGTC TTTGATTAAG CCCTTGTGTG AATATGACAA
1261 TGAGGAAAAA **GGAGATGCTT TTTTT**TTTTT TTAATCTTTT CATTGCTATA TTTATATGAA
1321 TGGTAGATAT ATTTGAATAT GAGATTTGCA ATTATTTATC ATTTCTGTTT CACTGATGTT
1381 GACTTCCTAA CATCAATGAT AAGGACTTGA ATTTGTGAAA TTATCTGCAT GTTGATTGAT
1441 GTCTTATTCC TAGTTTATAC TTGCATGCTA TAATTCTATA ATTATGTTGT TGATTGTGAC
1501 ATTGAGATGT CTTATGTGTT TTAAATTTTA A**GCAATGG**AG CTCGCTTTAT CCCTTGAGAA
1561 GCTAACAAAT GAAAAGTTAC TCCACTTGCA CAAGGTAGAG TTTTATTTAT GGCTTTCACA
1621 TTTTTGTTTA GGGTGTTGCT TGAGTTCTGA AGTTGTAGAA ATATACTTAT TATGTGCATT
1681 CTCTCTTAGG TGGCTGAAGA TAATCAAGAC GTGCAGATGA CCGAGTTTAT TGAAAGCGAA
1741 TTCTTGGGAG AACAGGTAAC AACGCCTCTG CGAGTGATTG ATTTTAATTA TACTTTGTTG
1801 TCACATAAGC ATAAATATTC TCAATGTTTA AAACTTAACC ATTCTAGATA GAAGCAATCA
1861 AGAAAATCTC AGAGTATGTT GCACAGCTAA GAAGACTAGG CAAGGGACAC GGTAAGGATA
1921 ACAACAATGC CCTCGCCCCC CATTGCCTCT TGGCTTTCGG TTTTCTCGTT CCTAATGTGT
1981 CTCCGGTGGT TAACGATATT CTTTTTGAAA CCTTGTTTTC AGGAGTGTGG CACTTTGATC
2041 AGATGTTGCT TCACGAGGAA GCATAAACAA AGGGATTTGG CTGCATAGAG TAAAAGTTTA
2101 GTGAAATATG AAAATAAAAT GGTGTCCAGG TTGGATGTGG TTGTTTGGAT TTCTAGTTTT
2161 AGAGGGCAGA TGATGATTTG TGTTAGAATT TTCTGTTGTT AACGTGTTAG TTAATGTTGT
2221 GTTTTGGTTT TCAAAGGTTT ATTTTGATGT TAATGGAAGA TTCTCACTTG TTTTTAATTC
2281 TAAACTAGAC TTTATTTACC CTATTT

Figure S2

Predicted coding sequence of *Cucumis sativus* ferritin protein XP004148174 (reported at NCBI as ferritin-3, chloroplastic-like, transcript variant 2 (LOC101221012). Exons are highlighted in yellow; position of primers Cucsafor1, Cucsarev1 and Cucsarev2 is underlined; position of primer Cucsarev3 is underlined in bold.
